# Prevalence of borrowing and sharing prescription medicines and associated socio-demographic factors: findings from COBERS health centres in northern Uganda

**DOI:** 10.1186/s40360-018-0206-5

**Published:** 2018-04-18

**Authors:** James Henry Obol, Peter Akera, Pamela Ochola Atim, Sylvia Awor, Ronald Wanyama, Kenneth Luryama Moi, Bongomin Bodo, Patrick Olwedo Odong, Emmanuel Otto Omony, Hussein Oria, David Musoke, Felix Kaducu

**Affiliations:** 1 Department of Public Health, Faculty of Medicine, Gulu University, P.O Box 166, Gulu, Uganda; 2 Department of Obstetrics and Gynaecology, Faculty of Medicine, Gulu University, P.O Box 166, Gulu, Uganda; 3 Department of Biochemistry, Faculty of Medicine, Gulu University, P.O Box 166, Gulu, Uganda; 4 Department of Medical Microbiology & Immunology, Faculty of Medicine, Gulu University, P.O Box 166, Gulu, Uganda; 5 Department of Paediatrics and Child Health: Faculty of Medicine, Gulu University, P.O Box 166, Gulu, Uganda; 6 District Health Office, Amuru District Local Government, P.O Box 1074, Gulu, Uganda; 7 District Health Office, Agago District Local Government, P.O Box 1, Agago, Uganda; 8 Department of Pharmacy, School of health Sciences Makerere University, P.O Box 7072, Kampala, Uganda; 9 Department of Pharmacology, Faculty of Medicine, Gulu University, P.O Box 166, Gulu, Uganda

**Keywords:** Socio-demographic factors, Borrowing and sharing prescription medicines, COBERS health centres, Gulu University

## Abstract

**Background:**

The use of prescription medications without the involvement of medical professionals is a growing public health concern. Therefore this study was conducted to determine the prevalence of borrowing and sharing prescription medicines and associated socio-demographic factors among community members who had sought health care from COBERS health centres.

**Methods:**

We conducted analytical cross – sectional study among former patients who sought treatment during the two months period prior to data collection in nine COBERS health centres. We used cluster proportional-to-size sampling method to get the numbers of research participants to be selected for interview from each COBERS site and logistic regression model was used to assess the associations.

**Results:**

The prevalence of borrowing prescription medication was found to be 35.9% (95% CI 33.5–38.2%) and sharing prescription medication was 32.7% (95% CI 30.4–34.9%). The Socio-demographic factors associated with borrowing prescription medicines were: age group ≤19 years (AOR = 2.64, 95%CI 1.47–4.74, *p*-value = 0.001); age group 20–29 years (AOR = 2.78, 95%CI 1.71–4.50, *p*-value≤0.001); age group 30–39 years (AOR = 1.90, 95%CI 1.18–3.06, *p*-value = 0.009); age group 40–49 (AOR = 1.83, 95%CI 1.15–2.92, *p*-value = 0.011); being a female (AOR = 2.01, 1.58–2.55, *p*-value< 0.001); being a Pentecostal by faith (AOR = 1.69, 95%CI 1.02–2.81, *p*-value = 0.042) and being Employed Salary Earner (AOR = 0.44, 95%CI 0.25–0.78, *p*-value = 0.005). The socio-demographic factors associated with sharing prescription medicines were: age group ≥19 years (AOR = 4.17, 95%CI 2.24–7.76, *p*-value< 0.001); age group 20–29 years (AOR = 3.91, 95%CI 2.46–6.29, *p*-value< 0.001); age group 30–39 years (AOR = 2.94, 95%CI 2.05–4.21, *p*-value< 0.001); age group 40–49 years (AOR = 2.22, 95%CI 1.29–3.82, *p*-value = 0.004); being female (AOR = 2.50, 95%CI 1.70–3.47, *p*-value< 0.001); being Pentecostal by faith (AOR = 2.15, 95%CI 1.15–4.03, *p*-value = 0.017); and being engaged in business (AOR = 1.80, 95%CI 1.16–2.80, *p*-value = 0.009).

**Conclusion:**

A high proportion of study participants had borrowed or shared prescription medicines during the two months prior to our study. It is recommended that stakeholders sensitise the community members on the danger of borrowing and sharing prescription medicines to avert the practice.

**Electronic supplementary material:**

The online version of this article (10.1186/s40360-018-0206-5) contains supplementary material, which is available to authorized users.

## Introduction

### Background

The use of prescription medications without the involvement of medical professionals is a growing public health concern [1, 2]. Borrowing prescription medication is when a patient takes medication which is prescribed for someone else [3, 4] and sharing is a situation of giving one’s medications to someone [2]. In our study, borrowing prescription medicine means receiving the medicine with the implied intention of returning the same while sharing prescription medicine is giving part of one’s medicine to someone else.

This behaviour is of medical and public health concern because of the many potential adverse consequences [5, 6]. Some of the adverse consequences are resistance to the medicine, wrong dosing or duration of treatment leading to delay in cure of a condition thereby making someone believe that the treatment was ineffective. There is also a likelihood of some adverse events occurring as the result of using the medication [2, 3, 7, 8].

In developed countries, studies have investigated the influence of demographic factors on the rate of medication borrowing. Studies have shown that women share medications more than men but both men and women were likely to borrow medications equally [3, 9]. In Africa, there is little information on the prevalence of prescription medication borrowing and sharing as well as associated socio-demographic factors [3, 9]. Research has shown that the prevalence of prescription medication borrowing increases through adolescence, it peaks in the third decade and then decreases as age increases [4].

Study has shown that the most frequently borrowed prescription medications are opiates and hypnotics [3]. Analgesics had a high lifetime rate at 35% of being borrowed [10]. Results from other researches show that other prescription medications borrowed, were for conditions such as acne, allergy, pain, birth control, asthma amongst others [11, 12].

In developing countries, people are more likely to borrow, share prescription medicine because of the large stock of medicines kept at home for reuse, or given to those who request them [13]. The prevalence and socio-demographic factors that are associated with prescription medication borrowing and sharing in Uganda is scanty. The Uganda Ministry of Health in its 2010 Annual Health Sector Performance Report noted that there had been inherent drug stock out at the health facilities in Uganda [14]. Northern Uganda is just recovering from over two decades of armed conflict, which has led to breakdown of healthcare and social services. The poverty rate is 43.7% while the national average is 19.7% [15]. In the light of the above, we then undertook to explore whether the community members practice the habit of sharing or borrowing prescription medicine; what types of prescription medicines are the most commonly borrowed or shared; and their sources. The information gathered will provide the basis for actions by stakeholders (The Ministry of Health, the Local Government, the Development partners and Gulu University Faculty of Medicine) to combat these habits, which have potentially negative consequences on individual, and community health. Therefore, this study aimed at determining the prevalence of borrowing and sharing prescription medicines as well as associated socio-demographic factors among community members who sought health care from the health centres used for Community Based Education Research and Services (COBERS) by Gulu University. COBERS is an academic program during which 4th year medical students are attached to lower health centres for six (6) weeks every semester. During the COBERS placement, medical students participate in both preventive and curative healthcare services as well as community outreach. Therefore, our findings would help in public health campaign to mitigate the adverse impact of borrowing and sharing prescription medicine in the society. The results would also help to create awareness among all health care professionals in finding out whether the patient has had any medicine prior to coming to the health centre for treatment so that correct dosing can be provided.

## Methods

### Study design

This was a cross sectional analytical study conducted in March 2014.

### Study setting

Gulu University uses 11 health centres for COBERS as part of community attachment by medical students during their fourth year of study. We used nine (9) COBERS sites in this study of which five (5) are health centre levels III and four (4) are health centre level IV. Health centre IIIs and IVs provide both curative and preventive services. The health centres also supervise and support planning and implementation of services by the lower health units that are under their areas of jurisdiction [16].

### Study population

The study population were former patients who had sought medical care at the health centres two months prior to data collection. All patients who had received healthcare services from the selected COBERS sites in the last two months before data collection were eligible to participate in the study. We excluded patients referred from other health facilities; those with unknown address; and those who had died.

### Sample size estimation and sampling procedure

We used the modified Kish Leslie formula of 1965 [17] to estimate our sample size. Since the proportion of borrowing and sharing prescription medicines were unknown, we used 50% as our proportion of patients borrowing and sharing prescription medicines. The desired level of precision was set at 5% with the standard normal deviate of 1.96 and a design effect of 2.0 to give sample size of 769 in each of the two months. We assumed that 10% of the participants would not consent/assent to be in the study and thus we selected 1692 eligible study participants of which 78 potential participants did not consent/assent were not included in the study.

We purposively selected COBERS health centres because medical students use them during community placement and, using simple random sampling technique without replacement, we selected nine (9) out of eleven (11) health centres as clusters. We used data capturing form to extract information about former patient’s addresses from the health management information system (HMIS) of the health centres. We used the list as our sampling frame. We then used cluster proportional to size sampling method to get the number of research participants to be selected for interview from each COBERS site. After we had obtained the numbers of study participants in each cluster, we used simple random sampling technique without replacement to generate research participants for the interview. Using the former patients’ bio data, research assistants traced the eligible participants to their homes and interviewed them using a semi-structured questionnaire.

### Data collection, quality and data management

We used semi-structure questionnaires with both open-ended questions and closed ended questions (Additional file 1), which research assistants delivered to the respondents, and recorded their responses. The questionnaire was translated into the local language (Acholi) and then back translated into English to ensure that the meaning of the questions were not lost during translation. We recruited six research assistants whom we trained. During the training, we tested the questionnaire in two villages, which were not part of the communities served by the COBERS health centres to minimise errors during data collection. Close monitoring of data collection process was done to ensure that the questionnaires were filled correctly. Data was double entered into Epidata version 3.1, backed up, edited and cleaned by the researcher to ensure data quality.

### Data analysis

The data was exported to STATA version 11 (StataCorp, College Station, Texas 77,845 USA) for analysis. Univariate analysis was performed for socio-demographic characteristics and prevalence of borrowing and sharing prescription medicines. Bivariate analysis was done using Chi-squared test to assess for association between the socio-demographic characteristics and prescription medication borrowing/sharing among participants. We used logistic regression model to assess for association between socio-demographic characteristics and borrowing/sharing prescription medicines. We calculated cluster-adjusted odds ratios plus 95% confidence intervals (CI) for the independent variables. We adjusted for clustering of data among communities around each COBERS site. Any socio-demographic characteristic at multivariate analysis with *P*-value ≤0.05 was taken as a significant predictor of borrowing and sharing prescription medicine.

## Results

The age of the study participants ranged from 12 to 87 years with median of 31 years with inter-quartile range of 25–42 years. The majority of the participants were in the age range 20 to 29 years; were females; were at primary education level only; were Catholics; were married or cohabiting; and were working as peasant farmers. The factors associated with borrowing prescription medicines at bivariate were:- age groups of participants, sex, religious affiliation and occupation. The factors associated with sharing prescription medicine among participants at bivariate were-age groups, sex, religious affiliation and occupation. Table 1 summarises the socio-demographic factors and chi-square test results for borrowing and sharing prescription medicine.

**Table 1:** Socio-demographic factors and Chi-squared test results for borrowing and sharing prescription medicine, *n* = 1614

	Frequency	Borrowed Prescription Medicine				Shared Prescription medicine			
Variable	n (%)	Yes (%)	No (%)	Χ^2^	*P*-value	Yes (%)	No (%)	Χ^2^	*P*-value
Age groups									
60 and above years	117 (7.2)	27 (23.1)	90 (76.9)	22.17	< 0.001	17 (14.5)	100 (85.5)	44.94	< 0.001
≤ 19 Years	105 (6.5)	43 (41.0)	62 (59.1)			42 (40.0)	63 (60.0)		
20–29 years	532 (33.0)	225 (42.3)	307 (57.7)			216 (40.6)	316 (59.4)		
30–39 years	489 (30.3)	163 (33.3)	326 (66.7)			158 (32.3)	331 (67.7)		
40–49 years	236 (14.6)	78 (33.0)	158 (67.0)			63 (26.7)	173 (73.3)		
50–59 years	135 (8.4)	43 (31.9)	92 (68.2)			31 (23.0)	104 (77.0)		
Sex									
Male	512 (31.7)	124 (24.2)	388 (75.8)	44.28	< 0.001	103 (20.1)	409 (79.9)	53.58	< 0.001
Female	1102 (68.3)	455 (41.3)	647 (58.7)			424 (38.5)	678 (61.5)		
Level of education									
None	377 (23.3)	133 (35.3)	244 (64.7)	9.26	0.056	108 (28.7)	269 (71.4)	7.41	0.119
Primary education	786 (48.7)	305 (38.8)	481 (61.2)			268 (34.1)	518 (65.9)		
Ordinary level education	345 (21.4)	111 (32.2)	234 (67.8)			122 (35.4)	223 (64.6)		
Advanced level education	87 (05.4)	27 (31.0)	60 (69)			26 (29.9)	61 (70.1)		
Tertiary education	19 (01.2)	3 (15.8)	16 (84.2)			3 (15.8)	16 (84.2)		
Religious affiliation									
Catholic	940 (58.2)	325 (34.6)	615 (65.4)	13.09	0.009	290 (30.9)	650 (69.1)	20.71	< 0.001
Protestant	590 (36.6)	221 (37.5)	369 (62.5)			200 (33.9)	390 (66.1)		
Muslim	12 (0.8)	3 (25.0)	9 (75.0)			4 (33.3)	8 (66.7)		
Seventh Day Adventist (SDA)	15 (0.9)	1 (6.7)	14 (93.3)			1 (6.7)	14 (93.3)		
Pentecostal	57 (03.5)	29 (50.9)	28 (49.1			32 (56.1)	25 (43.9)		
Occupation									
Peasant farmer	1149 (71.2)	426 (37.1)	723 (62.9)	24.27	< 0.001	358 (31.2)	791 (68.8)	42.28	< 0.001
Employed Salary Earning	171 (10.6)	33 (19.3)	138 (80.7)			32 (18.7)	139 (81.3)		
Business	294 (18.2)	120 (40.8)	174 (59.2)			137 (46.6)	157 (53.4)		
Marital status									
Single/Widow/Divorce/Widower	362 (22.4)	122 (33.7)	240 (66.3)	0.96	0.351	108 (29.8)	254 (70.2)	1.68	0.204
Married/Cohabiting	1252 (77.6)	457 (36.5)	795 (63.5)			419 (33.5)	833 (66.5)		

35.9% (95% CI 33.5–38.2%) of the study participants had borrowed prescription medicines during the study period while 32.7% (95% CI 30.4–34.9%) of the study participants had shared their prescription medicine during the study period. The details are shown in Table 2.

**Table 2:** Prevalence of borrowing and sharing prescription medicine

Borrowed prescription medicine	Frequency	Percentage	95% CI
No	1035	64.1	
Yes	579	35.9	33.5–38.2%
Shared prescription medicine			
No	1087	67.3	
Yes	527	32.7	30.4–34.9%

Analgesics (pain killers) and anti-malarial drugs were the most commonly borrowed and shared prescription medicines. The details are shown in Table 3.

**Table 3:** Types of prescription medicine borrowed or shared (Multiple responses allowed)

	Borrowed Prescription Medicine		Shared Prescription Medicine	
Types of medicine	Frequency (n)	Percentage (%)	Frequency (n)	Percentage (%)
Analgesics (pain killers)	451	59.8	463	49.8
Anti-malarial drug	231	30.7	262	28.2
Antibiotics	71	9.4	196	21.1
Antiretroviral	1	0.1	2	0.2
Anti-helminthes	0	0.0	1	0.1
Allergies	0	0.0	6	0.6
Total	754	100.0	930	100.0

The most common sources of borrowed or shared prescription medicine were neighbours and family members. The details are shown in Table 4.

**Table 4:** Sources of borrowed and shared out prescription medicine (Multiple responses allowed)

	Borrowed from		Shared with	
Sources	Frequency (n)	Percentage (%)	Frequency (n)	Percentage (%)
Neighbours	435	75.1	338	62.9
Family members	119	20.6	182	33.9
Friends	14	2.4	17	3.2
Village Health Team members	8	1.4	0	0.0
Health workers	1	0.2	0	0.0
Workmates	1	0.2	0	0.0
Fellow HIV-positive patients	1	0.2	0	0.0
Total	579	100.0	537	100.0

The socio-demographic factors associated with borrowing prescription medicines were: age groups; being female and being a member of the Pentecostal faith. The socio-demographic factors associated with sharing prescription medicines were: age groups; being female; being a member of the Pentecostal faith and being a businessperson. The details are shown in Table 5.

**Table 5:** Multivariate analysis of socio-demographic factors associated with borrowing and sharing prescription medicine

	Frequency	Borrow Prescription Medicine			Share Prescription medicine		
Variable	n (%)	AOR	95% CI	*P*-value	AOR	95% CI	*P*-value
Age groups							
60 and above years	117 (7.2)	1.00			1.00		
≤ 19 Years	105 (6.5)	2.64	1.47–4.74	0.001	4.17	2.24–7.76	< 0.001
20–29 years	532 (33.0)	2.78	1.71–4.50	< 0.001	3.91	2.46–6.29	< 0.001
30–39 years	489 (30.3)	1.90	1.18–3.06	0.009	2.94	2.05–4.21	< 0.001
40–49 years	236 (14.6)	1.83	1.15–2.92	0.011	2.22	1.29–3.82	0.004
50–59 years	135 (8.4)	1.65	0.94–2.91	0.083	1.87	0.88–3.98	0.103
Sex							
Male	512 (31.7)	1.00			1.00		
Female	1102 (68.3)	2.01	1.58–2.55	< 0.001	2.50	1.70–3.47	< 0.001
Level of education							
None	377 (23.3)	1.00			1.00		
Primary education	786 (48.7)	0.94	0.69–1.27	0.676	0.92	0.73–1.15	0.442
Ordinary level secondary education	345 (21.4)	0.80	0.56–1.45	0.227	1.09	0.71–1.66	0.691
Advanced level high school education	87 (05.4)	0.94	0.51–1.74	0.840	0.98	0.63–1.53	0.946
Tertiary education	19 (01.2)	0.39	0.11–1.36	0.139	0.47	0.19–1.18	0.108
Religious affiliation							
Catholic	940 (58.2)	1.00			1.00		
Protestant	590 (36.6)	1.15	0.93–1.41	0.189	1.11	0.90–1.36	0.340
Muslim	12 (0.8)	0.58	0.16–2.09	0.404	0.89	0.34–2.30	0.807
SDA	15 (0.9)	0.12	0.01–1.09	0.060	0.12	0.01–1.47	0.096
Pentecostal	57 (03.5)	1.69	1.02–2.81	0.042	2.15	1.15–4.03	0.017
Occupation							
Peasant farmer	1149 (71.2)	1.00			1.00		
Employed Salary Earning	171 (10.6)	0.44	0.25–0.78	0.005	0.51	0.23–1.09	0.083
Businessperson	294 (18.2)	1.12	0.84–1.50	0.428	1.80	1.16–2.80	0.009
Marital status							
Single	362 (22.4)	1.00			1.00		
Married/Cohabiting	1252 (77.6)	1.14	0.87–1.47	0.340	1.17	0.87–1.57	0.311

*AOR* Adjusted Odds Ratio, *Single* Single/Widow/Divorce/Widower, *SDA* Seventh Day Adventist

## Discussion

The age of our study participants ranged from 12 to 87 years with the median age of 31 years. The results of our study showed that the prevalence of borrowing prescription medication was high with 35.9% of the respondents reported to have ever borrowed prescription medicines during the study period. Similarly, the prevalence of sharing prescription medicine was found to be high with 32.7% of the study participants reported to have ever shared prescription medicine during the study period. A systematic review of literature by Beyene et al. shows that the prevalence of prescription medication borrowing ranges from 5%, which is much lower, compared with the result of our study to 51.9%, which is much higher compared with the result of our study [18]. The review further shows that the prevalence of sharing prescription medicine ranges from 6% to 22.9%, which is much lower, compared with result of our study [18]. The prevalence rates of borrowing and sharing prescription medication in this study are 35.9% and 32.7% respectively. This falls in the range of systematic review that reports it from 5 to 51%. However, previous reports from developed countries show the rate of borrowing and sharing to be 25–26% and 22–24% respectively [3, 19, 20]. This could be due to convenience in accessing those medicines or lack of access to medical care [1].

Our study indicated a much higher prevalence of borrowing and sharing prescription medicine than the findings of most of the above studies. This could be due to inherent drug stocks out at the health facilities as was reported by the Uganda Ministry of Health in its 2010 Annual Health Sector Performance Report [14]. So people would tend to look for medicine from other sources to treat their conditions. In addition, Northern Uganda has been consistently ranked the poorest region in the country in terms of development index because of the over two decades of armed rebellion in the area. This could explain why they borrowed or shared prescription medicine because most people would not afford to pay for health care services in the private health facilities [15].

Medication for relieving pain was the most frequently borrowed and shared prescription medicine and this is consistent with other previous studies [1, 3, 9, 21, 22]. This could be because pain is a symptom that is felt by the patient and it may negatively affect the patient’s quality of life from day to day. The second frequently borrowed and shared prescription medicine was anti-malarial medicine. Malaria is ranked number one among the top ten causes of morbidity and mortality for all age groups in Uganda [16]. It usually presents with headache in people who are infected. From the patient’s perspective therefore, prompt treatment with anti-malaria medicine and pain reliever is vital for patients’ recovery and relief. This could explain why pain relievers and anti-malarial medicine were the most frequently borrowed and shared prescription medicine. Neighbours, family members and friends were the most common sources of borrowed and shared prescription medicine. This is consistent with other previous research findings from elsewhere [18, 19].

### Socio-demographic factors associated with borrowing and sharing prescription medication

In our study, age was found to be significantly associated with borrowing and sharing prescription medicine. The prevalence of borrowing and sharing prescription medicine peaked at the age group 20 to 29 years and then declined with increasing age. This could be due to poverty and the change in life style on becoming independent within the community making people more exposed to diseases such as malaria. Because of high poverty rate, most people are unable to pay for medical expenses in privates’ clinic. The government health centres which offer free services always suffer from frequent drug stock outs. Our finding is in contrast with other studies which showed that the prevalence of medication borrowing increases through adolescence, peak during the third decade of life, and then generally declines with increasing age [1, 4].

Furthermore, women are twice as likely to both borrow and share prescription medication compared to men. This agrees with the findings of other previous studies [1, 3, 9, 23]. Research participants who were members of the Pentecostal faith were 1.69 times more likely to borrow prescription medicine than those from other religious faiths (*p*-value = 0.042). They were slightly over two times more likely to share prescription medication than those from other religious faiths (*p*-value = 0.017). This interesting finding requires further investigation. The question arises whether the Pentecostal preaching of sharing or their belief that prohibits the use of Western medicine drives them to borrow and share prescription medicine so as not to people do not see them at health facilities when sick.

The research participants who were gainfully employed and earning salary were 0.44 times less likely to borrow prescription medicine than other participants (*p*-value = 0.005). This could be because employed salary earning persons are able to pay for healthcare services and transport themselves to the nearest health facility in case of emergency. In addition, those people who are employed are literate and tend to be cautious in making decision related to their health. Therefore, they would be less likely to borrow prescription medicine. In addition, job related engagements would make them busy and interact less with other members of the community. Being somewhat detached from the community would make them less likely to borrow prescription medicine in case of need.

Participants who were engaged in business were 1.8 times more likely to share their prescription medicine than other research participants who were either gainfully employed earning salary or peasant farmers (*p*-value = 0.009). This could be because being engaged in business enables one to afford to pay for medical services including prescription medicine and would be willing to help those in need so as to be seen as someone who cares for others in the society.

Our study had some limitations. The study was a cross-sectional survey and so we could not reliably estimate the pattern of prescription medication borrowing and sharing since there could be seasonal variations. Furthermore, because of the cross-sectional nature of the study, we could not draw cause and effect relationship and our results were limited to association only. It should be noted that, although the prevalence of prescription medication borrowing and sharing were high compared to other studies done elsewhere, social desirability bias might have resulted in underreporting resulting in underestimate of the true prevalence of prescription medication borrowing and sharing in the study population especially with regards to antiretroviral (ARV) medicines. Also, we did not probe those who responded that they neither borrowed nor shared prescription medicine if they would consider lending medication or borrowing it themselves should the need arise; or whether they refrain from borrowing and sharing because they were aware of the potential hazards.

Our study has several important inferences among which are that it is a community-based study. It is the first study in Uganda that utilises members of the community who had sought health care from health centres within their locality. The prevalence of prescription medication borrowing and sharing was significantly high in our study. About one in every three-research participants had reportedly borrowed or shared prescription medicine during the study period. This is of particular importance to medical professionals as it provides insight into the prevalence of prescription medication borrowing and sharing among community members who use COBERS health centres for medical care. With these findings, there is need for providers of medical care to always probe for borrowing prescription medicine while taking patient’s medication history. This would help in prescribing medications since; in any given day, several of their patients might have borrowed prescription medication and used them irrationally before coming to the facility for treatment. Thus, there is need for medical care providers to always inquire about medication use, and sensitise the patients about the danger of prescription medication borrowing and sharing.

## Conclusions

About one third of the participants have borrowed or shared prescription medicine within the study period, which is a high proportion. Factors, which promote borrowing prescription medicine, were-being female, and ages below 50 years while being employed salary earner avert the habit. Factors associated with sharing prescription medicine were- being female, ages below 50 years and person’s occupation that is classified as business. Therefore, stakeholders should sensitise community members on the danger of borrowing and sharing prescription medicine so that the practice is averted. We encourage health care providers to always probe for borrowing prescription medicine while taking patient’s medication history. This can help in prescribing medications since, in any given day; several of their patients might have borrowed prescription medication and used them before coming to the facility for treatment. There is need for further study to determine the pattern of borrowing and sharing prescription medicine and the reasons for borrowing and sharing prescription medicine.

## Additional file

Additional file 1: Questionnaire for the survey. (DOC 44 kb)

## Abbreviations

AOR: Adjusted Odds Ratio
ARV: Antiretroviral
CI: Confidence Interval
COBERS: Community Based, Education, Research and Services
GU-REC: Gulu University Research Ethics Committee
SDA: Seventh Day Adventist
UNCST: Uganda National Council for Sciences and Technology
