# Supplementary material for: Prevalence of borrowing and sharing prescription medicines and associated socio-demographic factors: findings from COBERS health centres in northern Uganda
Source: BMC Pharmacol Toxicol. 2018 Apr 18;19:17. doi: 10.1186/s40360-018-0206-5 (PMC5907402; doi:10.1186/s40360-018-0206-5)
Supplement: Supplementary file 1 — Questionnaire for the survey. (DOC 44 kb) [file 40360_2018_206_MOESM1_ESM.doc]

**Appendix V: Mapping Distances in Accessing Healthcare Services and Common health Problems Reported at COBERS Sites by Communities Questionnaires**

**IDENTIFICATION**

Interviewee Name........................................................................................................

Village Name……………………………………Parish Name…………………………………

Sub-county Name……………………………...District Name…………………..................…

Date............./........../..............(dd/mm/yyyy) Interviewer’s Name……………………………

Result of Questionnaire

1. Completed
2. Refused
3. Partly Completed

Supervisor’s Verification

1. Yes
2. No

**Questions: Socio-demographic characteristics**

1. How old are you now?............................Completed Years
2. When were you born? (1). .........../........../........(dd/mm/yyyy) (2) Don’t know
3. Sex 1. Male 2. Female
4. What is your level of education? 1 None 2 Primary 3 O level 5 A level 4 Tertiary 5 University
5. Religion: 1 Catholic 2 Protestant 3 Muslim 4 SDA 5 Pentecostal
6. Are you currently employed or earning an income? 1 Yes 2 No
7. Occupation: 1 Peasant farmer 2 Employed Salary Earning 3 Business
8. Marital status: 1 Single 2 Married 3 Cohabiting 4 Widow 5 Divorced 6 Widower
9. Number of people in your household...........................................
10. Does your household have a pit latrine/toilet being used currently? 1 Yes 2 No
11. If no in the above, where do you ease yourself (defecate)? 1 Share pit latrine with neighbour 2 Use bushes behind the house/homestead 3 Dig and burry
12. What water source is your household currently fetching for household use? 1 Borehole 2 Protected well/spring 3 Pond/dam 4 Stream/river

**Questions: Accessibility of Healthcare Services**

1. How far is the health Center from your home?.........................KM
2. How long does it take you to travel to the health centre?..........................Hours
3. How do you normally travel to the health centre when you are sick? 1. Footing 2. Bicycle 3. Motor cycle 4. Motor car
4. Have you ever gone to the health centre when you were sick and the health worker told you that there was no medicine for your sickness? 1. Yes 2. No
5. If yes in the above, what did you do? 1. Did nothing 2. Bought from drug shop/clinic 3. Share the one from relative/friend 4. Borrowed from a neighbour
6. When medical students are available, we get services free of charge? 1. Yes 2. No
7. Health workers always refer us to buy medicine. 1. Yes 2. No
8. I am able to get medical care when medical students are available. 1 Yes 2. No 3 Not sure
9. What time are services available at the health centre? 1 Opening …….…. 2 closing …………
10. On average, how long did you have to wait before getting to see the health worker …………. (Minutes)

**Common Health Problems**

1. When you went to the health centres, what was your main complaint or illness?

Fever/malaria ...............1

Cough/chest infection ...2

Tuberculosis .................3

Asthma .........................4

Headache .....................5

Diarrhea ........................6

Vomiting ........................7

Stomach pain ................8

Skin problem .................9

Ear ............................... 10

Eye problem ................ 11

Hypertension ............... 12

Diabetes ...................... 13

Pregnancy related ....... 14

Delivery related ............ 15

HIV/AIDS ..................... 16

Injury ............................ 17

Other ............................ 18

1. Did the health worker see you for the condition(s) you had in the above? 1 Yes 2 No
2. If yes in the above, what did the health worker do for the complaint or illness you had? 1 Nothing 2 Prescribed Medicine 3 Referral
3. If the health worker prescribed medicine in above, did you get the medicine for the compliant or illness you presented with? 1 Yes 2 No
4. If you did not get medicine in the above, what did you do? 1 Returned home & went back later to the health facility 2 Bought medicine from drug shop 3 Returned home & use herbs
5. If 2 more conditions presented with (in 23) did you get treatment for all conditions you presented with? 1 Yes 2 No
6. List the most common illnesses in this area. (i)……………………………………..

(ii)…………………………………………………(iii)…………………………………………

(iv)…………………………………………………(v)………………………………………..

1. In your opinion, what do you think the community can do to solve their own health problems you have listed above?.................................................................................

.................................................................................................................................

**Satisfaction with healthcare services**

1. How satisfied are you with the skill and competency of the students?

1 very dissatisfied 2 not satisfied 3 Satisfied 4 very satisfied

1. Medical students spent plenty of time while providing medical care to me. 1 Yes 2 No
2. How long was the consultation time during your last visit ………. Minutes
3. Health workers do not pay attention to me whenever I go to get treatment.

1 Yes 2 No

1. Where you treated in a friendly manner the last time you went to the health facility? 1 Yes 2 No
2. If no in 36 above, how were you treated?.........................................................................
3. How long on average do health workers takes to provide emergency services?…………… (Minutes)
4. When medical students are around, I feel confident that the medical care I need, I will get it without pay for anything 1 Yes 2 No
5. Medical students always explain my health problem and the treatment given? 1 Yes 2 No
6. Did the health worker explain your problem and treatment given? 1 Yes 2 No
7. What services did you receive the last time you went to the health centre? 1 Blood Pressure measured 2 Lab test 3 weight Taken 4 Temperature measured 5 Checking body/Physical examination 6 others specify …………………………………………………………………………………………………
8. Medical students at times ignore what I tell them? 1 Yes 2 No
9. I am very satisfied with the medical care I received before medical students came to the health centre? 1 Yes 2 No
10. When students are around, it is easy to get services during emergency? 1 Yes 2 No
11. Who normally makes the decisions about the healthcare of members of your household?……………………..
12. Have you ever been mistreated at the facility? 1 Yes 2 No
13. If yes in 47 above, by whom? 1 Nurse 2 Midwife 3 Clinical officers 4 Record officer 5 Medical students
14. What was the form of mistreatment in 47 above?.............................................................

……………………………………………………………………………………………………..

**Healthcare Seeking Practices**

1. When you or your family members fall sick, where do you go for treatment first? 1 Health facility 2 Clinic 3 Drug shop 4 Use herbal medicine
2. The last time you were sick, did you seek treatment before coming to the health centre? 1 Yes 2 No
3. If yes in above, from where?........................................................................................
4. Why did you go to that place first?................................................................................

………………………………………………………………………………………………….

1. Where do you commonly get medicines from if you or someone is sick in home?

1 Health facility 2 Drug shops 3 Clinic

**Collecting Medicine for later use**

1. Have you ever gone to the health centre to collect medicine when you were not sick? 1 Yes 2 No
2. If yes in above, why did you go to collect the medicine?...............................................

......................................................................................................................................

1. Have you ever borrowed prescription medication from others? 1 Yes 2 No
2. If yes in above from whom?..............................
3. What was the sickness and the medicine which you borrowed in 56 above?.............................................................................................................................

……………………………………………………………………………………………………..

1. Have you ever shared your prescription medication with others? 1 Yes 2 No
2. If yes in above, who did you share with?. ……………………..
3. List all the medicines which you have shared...................................................................

.........................................................................................................................................

1. How many times have you shared medicine in the past one year?..................Times
2. During the past year, have you had difficulty getting medical treatment? 1 Yes 2 No
3. During the past year, were you prescribed a medicine but were unable to get it? 1 Yes 2 No
4. In the past year, have you had difficulty understanding what your doctor or other healthcare professional was telling you? 1 Yes 2 No
5. Overall, how satisfied are you with the communication you have with your doctor or other healthcare staff related to your health? 1 very dissatisfied 2 not satisfied 3 Satisfied 4 very satisfied
